# Lactate Conversion by Lactate Dehydrogenase B Is Involved in Beige Adipocyte Differentiation and Thermogenesis in Mice

**DOI:** 10.3390/nu15224846

**Published:** 2023-11-20

**Authors:** Bin Guo, Hui Shu, Ling Luo, Xiangpeng Liu, Yue Ma, Jie Zhang, Zhiwei Liu, Yong Zhang, Lei Fu, Tongxing Song, Yixue Qiao, Chi Zhang

**Affiliations:** 1 The Tenth Affiliated Hospital of Southern Medical University (Dongguan People’s Hospital), Dongguan 523018, China; bzg18@smu.edu.cn; 2 Cambridge-Suda Genomic Resource Center, Soochow University, Suzhou 215123, China; shu_hui@suda.edu.cn (H.S.); 20224250011@stu.suda.edu.cn (L.L.); 20214250007@stu.suda.edu.cn (X.L.); mayue@suda.edu.cn (Y.M.); jiezhang@suda.edu.cn (J.Z.); zwliu@suda.edu.cn (Z.L.); yong.zhang@suda.edu.cn (Y.Z.); 3 Wisdom Lake Academy of Pharmacy, Xi’an Jiaotong-Liverpool University, Suzhou 215123, China; lei.fu@xjtlu.edu.cn; 4 College of Animal Science and Technology, Huazhong Agricultural University, Wuhan 430070, China; songtongxing@mail.hzau.edu.cn

**Keywords:** lactate dehydrogenase B (LDHB), beige adipocyte, thermogenesis

## Abstract

Adipose tissue (AT) is the primary reservoir of lipid, the major thermogenesis organ during cold exposure, and an important site for lactate production. However, the utilization of lactate as a metabolic substrate by adipocytes, as well as its potential involvement in the regulation of adipocyte thermogenesis, remain unappreciated. In vitro experiments using primary stromal vascular fraction preadipocytes isolated from mouse inguinal white adipose tissue (iWAT) revealed that lactate dehydrogenase B (LDHB), the key glycolytic enzyme that catalyzes the conversion of lactate to pyruvate, is upregulated during adipocyte differentiation, downregulated upon chronic cold stimulation, and regained after prolonged cold exposure. In addition, the global knockout of *Ldhb* significantly reduced the masses of iWAT and epididymal WAT (eWAT) and impeded the utilization of iWAT during cold exposure. In addition, *Ldhb* loss of function impaired the mitochondrial function of iWAT under cold conditions. Together, these findings uncover the involvement of LDHB in adipocyte differentiation and thermogenesis.

## 1. Introduction

Adipose tissue (AT) forms the inner energy reservoir of the body, where it stores triglycerides to meet metabolic demands, and possesses a remarkable capacity to respond to internal and external changes [1]. AT encompasses distinct types of adipocytes, namely white, beige, and brown adipocytes [2,3,4]. Dysfunction in adipocyte metabolism contributes to common systemic metabolic disorders like obese and type 2 diabetes (T2D) [5,6,7,8], as well as diseases that would be encumbered by a disorientation in metabolism, for example, heart failure [9]. Thus, it is important to understand the cellular metabolism of adipocytes in physiology and disease.

Adipocytes mainly utilize lipid and glucose as major energy substrates [10,11] to support them through different physiological and pathophysiological states [11,12]. In hypertrophic white adipocytes, defects of glucose metabolism were identified as a cardinal event in the development of insulin insensitivity as early as 1976 [7]. On the other hand, thermogenic adipocytes, namely brown and beige adipocytes, rapidly oxidize fatty acids and glucose to dissipate energy as heat during exposure to cold [13,14,15]. However, besides lipid and glucose, recent studies indicate the involvement of creatine metabolism in thermogenesis [16,17], providing a rationale to search for additional alternative energy substrates for adipocytes. Also, it is intriguing to explore the molecular mechanisms regulating the preferential utilization of various substrates.

Recently, the production of lactate, which is the best-known waste product of anaerobic metabolism [18,19,20], has received considerable attention as an important regulator of adipocyte metabolism [21,22]. Lactate is now widely acknowledged as the continuous fuel for different cells under completely aerobic conditions [23,24]. Indeed, adipocytes contribute substantially to the inter-cell and inter-organ fluxes of lactate [7,17,21,25]. However, the utilization of lactate by adipocytes themselves, the type(s) of adipocytes that could use lactate as fuel source, as well as the contribution of lactate utilization to AT metabolism and functions remain ambiguous and poorly understood. Lactate dehydrogenase B (LDHB) is a key glycolytic enzyme that catalyzes the conversion of lactate to pyruvate, which is the substrate used for mitochondrial adenosine triphosphate (ATP) production via tricarboxylic acid (TCA) cycle [26]. Emerging evidence shows that the induction of LDHB expression causes enhanced mitochondrial oxidative phosphorylation (OXPHOS) and sustains increased glycolysis [27,28]. These observations indicate that alterations in LDHB expression or function may remodel the cellular utilization of lactate and provide a rationale to explore the importance of LDHB in metabolic homeostasis.

The role of LDHB-dependent lactate conversion in adipocyte is barely understood. Here, using primary stromal vascular fraction (SVF) preadipocytes, isolated from mouse inguinal white adipose tissue (iWAT), wildtype C57BL/6N mice, and global *Ldhb* knockout mice (*Ldhb tm1a* −/−), compared to control littermates, we explored the possible function of LDHB in adipocyte metabolism. By inducing the differentiation process of primary iWAT SVF towards adipocytes, we found that LDHB is upregulated during adipogenic differentiation. In addition, as the ability to adapt to environmental temperature is a critical feature of adipocytes, we exposed iWAT SVF to cold. Western blotting analyses revealed that LDHB was downregulated upon cold stimulation and regained during prolonged cold exposure, implying an important role of LDHB in adipocyte thermogenesis. To explore the role of LDHB-dependent lactate conversion on the physiological state in vivo, we generated LDHB global knockout mice via the knockout-first method. The loss of functional LDHB significantly reduces the masses of iWAT and epididymal WAT (eWAT) in *Ldhb tm1a* −/− mice and impedes the utilization of iWAT during cold exposure. In addition, the proteins involved in OXPHOS are downregulated in the iWAT of *Ldhb tm1a* −/− mice after cold treatment, further supporting the involvement of LDHB in beige adipocyte thermogenesis. Together, these data identify an essential role for LDHB in the physiologic and cellular function of beige adipocytes in mice.

## 2. Materials and Methods

### 2.1. Animal Care

All experimental mice used in this study were of a C57BL/6N background and were bred and housed in the animal facility of the Cambridge-Suda Genomic Research Center at Soochow University (CAM-SU, Suzhou, China), with free access to acidified water and standard rodent chow food (radiated and autoclaved). Mouse maintenance and experimental use were performed according to animal protocols (ZJ-2021-1) for the usage of experimental mice, approved by the institutional Animal Care and Use Committee of CAM-SU on 24 December 2021.

*Ldhb* global knockout mice were generated using the knockout-first method. Briefly, blastocyst injections of mutant mouse embryonic stem (ES) cells, where cassettes containing mouse En2 SA, LacZ, Neo, FRT, and loxP sites were inserted in introns, were conducted by technicians at CAM-SU. Chimeric mice were then sequenced, and the positive insertion ones were bred with wild type C57BL/6N mice to obtain the *Ldhb Tm1a* mice. The sequences for the primers used for genotyping are listed in Table 1. Statistical analysis of embryo’s genotypes was performed, to evaluate the normality of birth.

The number of animals used in each experiment and the number in each group are stated in the figure legend section. In figures with individual data points, each point represents one mouse.

### 2.2. Preadipocyte Isolation and Adipogenic Differentiation In Vitro

Primary SVF preadipocytes were isolated from iWAT using a coalescent method of collagenase digestion and centrifugation separation. Briefly, iWAT from wildtype mice was minced and digested in digestion buffer containing 1.25 mg/mL of type I collagenase. The temperature was set at 37 °C during the digestion process to maximize the working efficiency of type I collagenase. The digestion was terminated after 40 min by adding the same volume of termination buffer (DMEM-based buffer with 20% FBS). After sufficient mixing, samples were centrifuged to remove the undigested tissues. Primary SVF preadipocytes were then isolated using centrifugation separation (1700 rpm for 5 min). The freshly isolated cells were cultured in a DMEM-based growth medium (GM) containing 20% FBS and 1% penicillin/streptomycin. The culturing environment was set at 37 °C with 5% CO_2_. For adipogenic differentiation, the GM was replaced by a DMEM-based induction medium (IM) containing 10% FBS, 2.85 mM insulin, 0.3 mM dexamethasone, 1 mM rosiglitazone, and 0.63 mM 3-isobutyl-methylxanthine, after cells reached a confluence of 100%. Cells were maintained in the IM for 4 days before switching to a DMEM-based differentiation medium (DM) containing 10% FBS, 200 nM insulin, and 10 nM T3.

### 2.3. Body Composition Measurement

The total body fat mass and lean mass of live mice were measured using a Minispec LF50 body composition analyzer without anesthesia. In brief, mice were placed in a clear plastic holder, which was then inserted into a tubular space on the side of the Minispec LF50 system. The mice were forced to keep still in the specially sized holder throughout the scan to ensure the accuracy of measurements.

### 2.4. Indirect Calorimetry Measurement

Oxygen consumption rate (VO_2_) and carbon dioxide production rate (VCO_2_) were measured using the indirect calorimetry system (Oxymax, Columbus Instruments, Columbus, OH, USA). Briefly, the indirect calorimetry system was kept in a temperature-stable environment (24 °C) with a 12-h light and 12-h dark cycle (light: 8 a.m.–8 p.m., dark: 8 p.m.–8 a.m.). During the whole process, mice were individually housed in each chamber, and were supplied with free access to food and water. Before the measurement, mice were allowed to adapt to the chamber for 24 h. Data in the present study were presented as uncorrected energy expenditure levels. The average energy expenditures of day (8 a.m.–8 p.m.) and night (8 p.m.–8 a.m.) were presented based on the average mean value of all numbers collected during the 12 h period.

### 2.5. Involuntary Treadmill Running

Before testing and data collection, we trained the mice for 5 days at a speed of 5 m/min for 5 min every day. Following the adaptation session, we forced the mice to run on a 15% incline, with an electric shock setting at a constant 0.7 mA in the indirect calorimetry program and the treadmill program at the same time. The treadmill program was designed with three sections: firstly, the speed was set as 5 m/min for 5 min; secondly, we increased the speed by 2.5 m/min every 2 min; after the speed reached 25 m/min, we forced the mice to run at 25 m/min for the last 4 min. After the treadmill program, we conducted the indirect calorimetry program with the exercised mice. All equipment was cleaned with 75% alcohol before and after use.

### 2.6. Cold Treatment

During cold-exposure experiments, the *Ldhb tm1a* −/− mice and the control littermates were singly caged and exposed to cold temperatures at 4 °C in an environmental chamber for 7 days. Control mice were maintained at room temperature in the same room.

### 2.7. Blood Biochemistry

A clinical chemistry analyzer (Hitachi 7100) was used to perform blood biochemistry analyses. The appropriate volume of blood that was required for the test (corresponding to 160–200 μL of plasma) was collected from each mouse and transferred to a gel tube containing lithium heparin. Samples were centrifuged for 15 min at the speed of 5000 rpm at 4 °C. Plasma samples were stored at −80 °C when analyses could not be performed immediately.

### 2.8. Total RNA Extraction and Real-Time Quantitative Reverse Transcription Polymerase Chain Reaction (qRT-PCR)

Total RNA was extracted using Trizol reagent, according to the manufacturer’s instructions. Following extraction, the purity of the RNA samples was checked using a spectrophotometer (Nanodrop 3000, Thermo Fisher Scientific, Waltham, MA, USA), and the acceptable ratio of absorption (260/280 nm) was set at 2.0. Three μg of RNA were then reverse transcribed using a HiScript III 1st Strand cDNA Synthesis Kit (Cat# R312, Vazyme, City of Dover, DE, USA), and the PCR program was set to 42 °C for 5 min, 37 °C for 15 min, and 85 °C for 5 min. Real-time qRT-PCR was carried out using a Roche Lightcycler 480 PCR System. SYBR Green Master Mix was used in this study. Primer sequences were retrieved from PrimerBank and are listed in Table 1. Relative changes in gene expression were calculated using the 2^−ΔΔCT^ method, and the expression level of mouse β-Actin was used as the internal control.

### 2.9. Western Blotting Analysis

Total protein extraction was performed using a radioimmunoprecipitation assay (RIPA) buffer (Beyotime, Haimen, China) supplied with protease inhibitor cocktail (Roche, IN, USA). Before Western blotting analysis, lysates were boiled in SDS sample buffer for 10 min and stored at −20 °C for further use. Proteins were separated using 10% SDS-PAGE and transferred to nitrocellulose membranes (Bio-Rad, Hercules, CA, USA), which were then blocked with 5% non-fat milk in TBST (Tris-buffered saline with 1% Tween) buffer at room temperature for 1 h. Membranes were incubated with the indicated primary antibody overnight at 4 °C, rinsed with TBST buffer, and incubated with the respective secondary antibody at room temperature for 1 h. Visualization was performed using enhanced chemiluminescence. Antibodies used in the present study are listed in Table 2.

### 2.10. Statistical Analysis

All analyses were conducted with Student’s *t*-test (two-tailed). All experimental data are presented as mean ± SEM. Comparisons with *p* values < 0.05, <0.01, or <0.001 were considered statistically significant.

## 3. Results

### 3.1. Lactate Dehydrogenase B Is Upregulated during Adipocyte Differentiation, Downregulated upon Cold Stimulation, and Regained during Prolonged Cold Exposure

**Figure 1:**
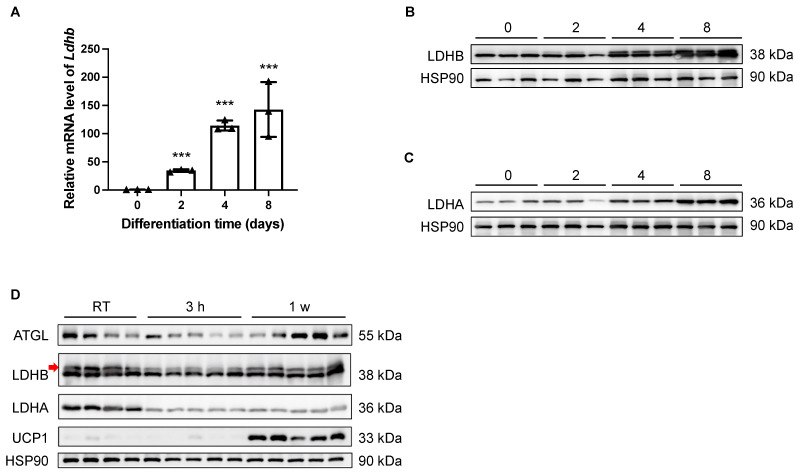
Expression levels of LDHB in iWAT were tightly controlled during adipocyte differentiation and thermogenesis. (**A**) Real-time qRT-PCR delta-delta CT (ddCT) of mRNA product of *Ldhb* in differentiated SVF preadipocytes isolated from iWAT at D0, D2, D4, and D8 (n = 4 animals used for primary cell isolation). Error bars show mean ± SEM. *** *p* < 0.001. (**B**,**C**) Western blotting analyses of differentiated SVF preadipocytes isolated from iWAT at D0, D2, D4, and D8, detecting LDHB (upper band, indicated with red arrow, (**B**)) and LDHA (**C**). HSP90 was used as loading control (n = 3 animals used for primary cell isolation). (**D**) Western blotting analyses of iWAT collected from treated or control wildtype C57BL/6N mice, detecting ATGL, LDHB (upper band, indicated with red arrow), LDHA, and UCP1. HSP90 was used as loading control (n = 4–5 animals used for iWAT collection).

Theoretically, LDHB is able to catalyze the interconversion between pyruvate and lactate in both directions; however, it preferably catalyzes the oxidation of lactate to pyruvate, sustaining the TCA cycle for energetic demands [26]. In light of the essential enzymatic role of LDHB, the potential involvement of LDHB in adipogenesis was examined in vitro. As shown by both qRT-PCR and Western blotting analyses, the expression levels of *Ldhb*/LDHB were dramatically increased in differentiated SVF preadipocytes isolated from iWAT at Day 2 (D2), D4, and D8, compared with the undifferentiated D0 control (Figure 1A,B). Similarly, the expression of LDHA was also elevated during the differentiation process of beige adipocytes (Figure 1C). Together, these data imply a potential role of LDHB in adipocyte differentiation. Next, we conducted prolonged cold-exposure experiments (1 w) to evaluate the involvement of LDHA and LDHB in the lipolysis and thermogenesis of iWAT, which are highly energetic processes. As the elevation in the uncoupling protein 1 (UCP1) expression level indicated [14,29], the cold-exposure experiments were properly performed (Figure 1D). At the onset of cold stimulation (3 h post-exposure), the protein levels of LDHA and LDHB dramatically decreased (Figure 1D). However, while the expression of LDHB recovered at the late stage of cold exposure, the reduction in LDHA level was sustained at 1 w (Figure 1D). These data suggest an essential role for LDHB in the lipolysis and thermogenesis of beige adipocytes.

### 3.2. Global Knockout of Lactate Dehydrogenase B Slightly Alters the Body Composition of Mice

As we observed a recovery of LDHB, but not LDHA, after a prolonged cold treatment (7 days, Figure 1D), we hypothesized that the usage of, but not the production of, lactate was important for the cold adaptation of beige adipocytes. We next injected mice with mutant mouse ES cells containing a modified *Ldhb* genome region to achieve *Ldhb* knockout (knockout-first). The *Ldhb* mutant mouse ES cells were retrieved from the ES cell bank of CAM-SU. Chimeric mice with a positive insertion were then bred with wild type C57BL/6N mice to obtain *Ldhb Tm1a* mice. The homozygous knockout alleles caused a premature translational stop and generation of a truncated peptide, lacking the key functional domain of LDHB.

**Figure 2:**
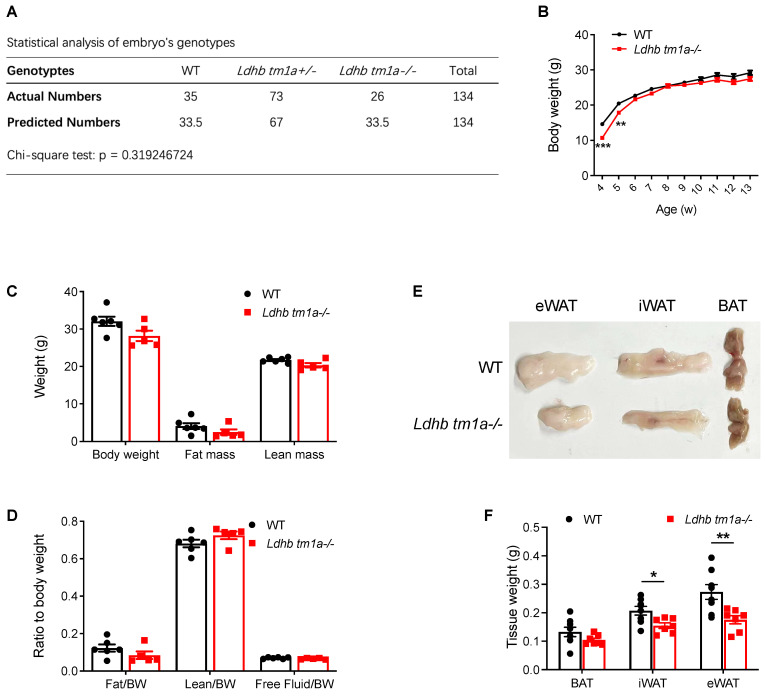
Global knockout of *Ldhb* significantly reduced the masses of iWAT and eWAT in *Ldhb tm1a* −/− mice. (**A**) Statistical analysis of embryos’ genotypes. (**B**) Body weights of *Ldhb tm1a* −/− mice or control littermates (WT) at indicated number of weeks of age (n = 6 or 14 for *Ldhb tm1a* −/− mice or WT, respectively). (**C**,**D**) Body composition of *Ldhb tm1a* −/− mice or control littermates (WT) at 11 w of age (n = 5 or 6 for *Ldhb tm1a* −/− mice or WT, respectively). (**E**,**F**) Representative gross views and quantification of brown adipose tissue (BAT), iWAT, and eWAT collected from *Ldhb tm1a* −/− mice or control littermates (WT) at 11 w of age (n = 7 or 8 for *Ldhb tm1a* −/− mice or WT, respectively). Error bars show mean ± SEM. * *p* < 0.05, ** *p* < 0.01, *** *p* < 0.001.

Mouse genotyping and statistical analysis based on numbers of littermates revealed that neither a heterozygous nor homozygous knockout of *Ldhb* affected the birth of mice (Figure 2A). Thus, we next analyzed the phenotype of homozygous knockout (*Ldhb tm1a* −/−) mice. At 4 and 5 w post-natal, *Ldhb tm1a* −/− mice exhibited a lower body weight than their control littermates (Figure 2B). However, the observed difference diminished at the later stage of their lifespan, which was after weaning (typically at 3–4 weeks of age) (Figure 2B). At 11 w of age, there were no significant differences in body weight or lean mass of WT and *Ldhb tm1a* −/− mice (Figure 2B–D). However, slight reductions in both fat mass and fat/body weight ratio were observed in *Ldhb tm1a* −/− mice (Figure 2C,D). Consistent with this, *Ldhb tm1a* −/− mice had smaller masses of iWAT and eWAT than the control littermates (Figure 2E,F). Together, these results indicate that the loss of *Ldhb* potentially causes abnormality in AT development during the early stage of postnatal growth, or enhanced utilization of fatty acids as fuel source.

### 3.3. Disruption of Lactate Dehydrogenase B Alters the Global Energy Metabolism of Mice at Nighttime

**Figure 3:**
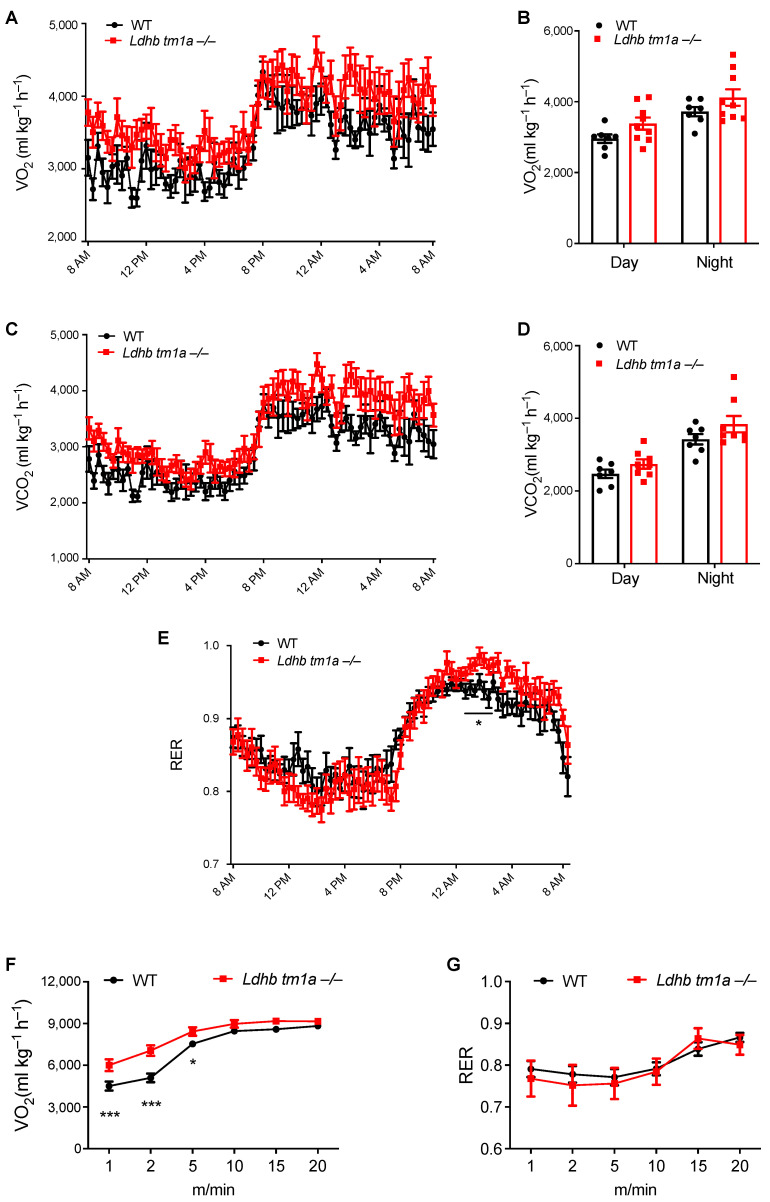
Disruption of *Ldhb*-altered global energy metabolism of mice at nighttime and during early stage of exercise. (**A**–**D**) The oxygen consumption (VO_2_, (**A**,**B**)) and carbon dioxide production (VCO_2_, (**C**,**D**)) of *Ldhb tm1a* −/− mice or control littermates (WT) (n = 9 or 7 for *Ldhb tm1a* −/− mice or WT, respectively). (**E**) RER of *Ldhb tm1a* −/− mice or control littermates (WT) were calculated based on data above (n = 9 or 7 for *Ldhb tm1a* −/− mice or WT, respectively). (**F**,**G**) The oxygen consumption (VO_2_, (**F**)) and RER (**G**) of *Ldhb tm1a* −/− mice or control littermates (WT) during running session (n = 13 or 10 for *Ldhb tm1a* −/− mice or WT, respectively). * *p* < 0.05, *** *p* < 0.001.

Given the importance of adipose tissue in energy supplementation for the whole body, we next examined how LDHB loss changes the systemic metabolism of mice. In the absence of forced exercise, while *Ldhb tm1a* −/− mice exhibited slight promotion in global energy metabolism, no statistically significant differences were observed in oxygen consumption (VO_2_) or carbon dioxide production (VCO_2_) (Figure 3A–D). When we calculated the respiratory exchange ratio (RER), *Ldhb tm1a* −/− mice had significantly elevated RER levels at nighttime (Figure 3E), implying their fuel preference towards carbohydrates over fatty acids for aerobic oxidation.

**Figure 4:**
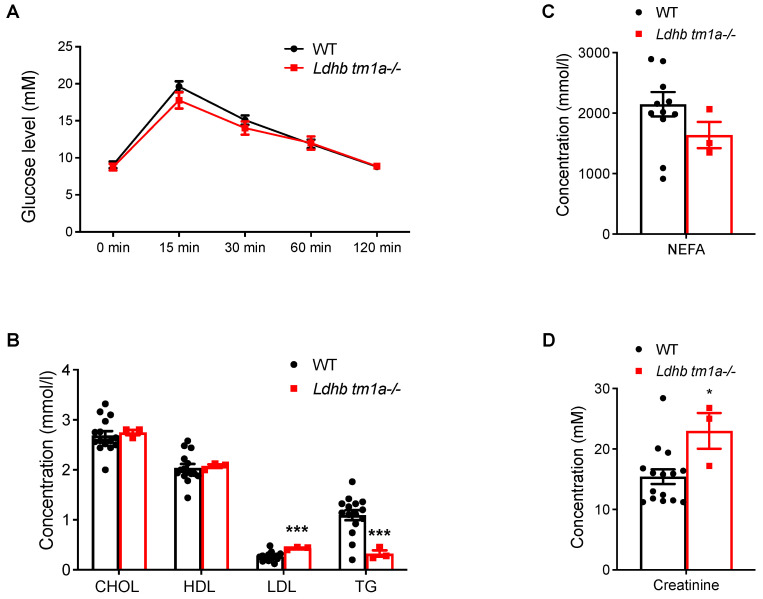
Global knockout of *Ldhb* did not alter responses to glucose. (**A**) Serum glucose levels of *Ldhb tm1a* −/− mice or control littermates (WT) during GTT (n = 10 for both groups). (**B**,**D**) Serum levels of CHOL, HDL, LDL, TG (**B**), NEFA (**C**), and creatinine (**D**) of *Ldhb tm1a* −/− mice or control littermates (WT) (n = 3 or 12–15 for *Ldhb tm1a* −/− mice or WT, respectively). * *p* < 0.05, *** *p* < 0.001.

The effect of *Ldhb* loss on the absorption and utilization of glucose were next studied, beginning with a glucose tolerance test (GTT). Comparing data collected from *Ldhb tm1a* −/− mice to those from the control littermates, no significant differences were observed in the initial glucose levels, glucose levels at 15 min post-injection, or glucose levels at the end of the evaluation, when the serum glucose level returned to normal (Figure 4A). Besides, the blood biochemistry tests showed that, while the concentrations of low-density lipoprotein cholesterol (LDL) were higher in *Ldhb tm1a* −/− mice, their serum concentrations of triglycerides (TG) and non-esterified fatty acids (NEFA) exhibited significant reductions (Figure 4B). Interestingly, *Ldhb tm1a* −/− mice had higher serum levels of creatinine (Figure 4D), an alternative fuel source for the body, and calcium (Ca^2+^, Supplemental Figure S1B).

### 3.4. Lactate Dehydrogenase B Loss Elevates Oxygen Consumption in Mice during the Early Stage of Involuntary Running

We next forced mice to run on a treadmill to evaluate the effect of LDHB loss on their global energy metabolism. At the onset of the experiment, the mice were allowed to run at a constant speed (5 m/min) for 5 min, following which the speed was increased by a rate of 2.5 m/min every 2 min. At the end of the acceleration phase, mice were forced to run at 25 m/min for 4 min. During the first 5 min of involuntary running, where anaerobic glycolysis was preferably utilized to provide energy over the aerobic oxidation of carbohydrates or fatty acids, *Ldhb tm1a* −/− mice consumed oxygen faster than the control littermates (Figure 3F), while their RERs remained the same (Figure 3G). These data suggest an impediment in anaerobic glycolysis or a preference for aerobic oxidation in *Ldhb tm1a* −/− mice during the early stage of involuntary running.

### 3.5. Lactate Dehydrogenase B Loss Impedes the Utilization of Inguinal White Adipose Tissue under Cold Exposure

**Figure 5:**
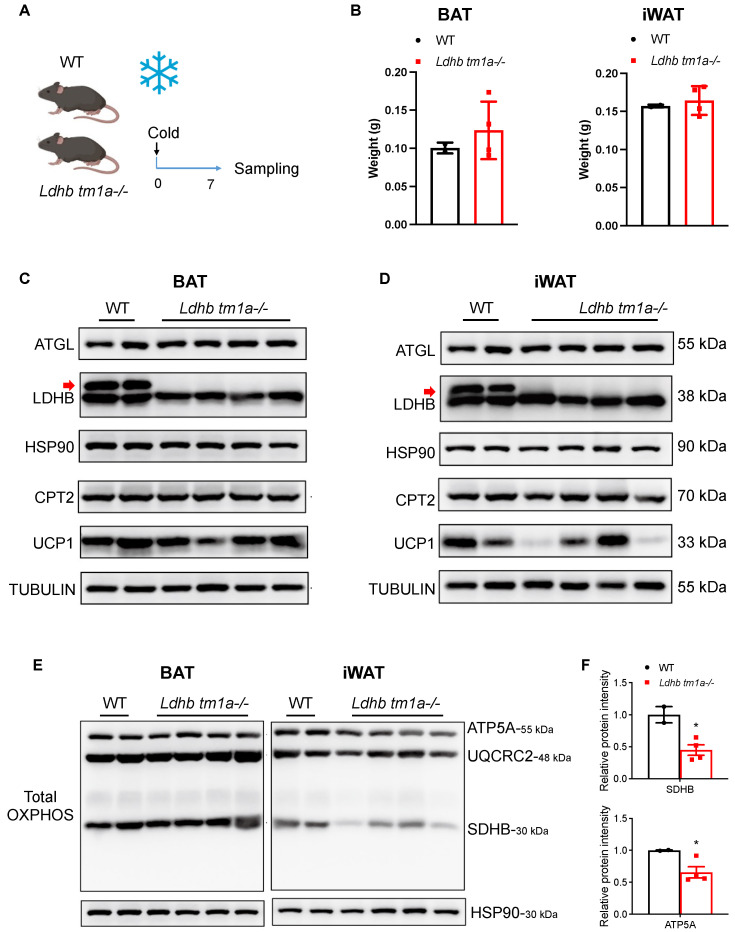
*Ldhb* loss impeded the utilization of iWAT under cold exposure. (**A**) Schematic illustrating the cold exposure experimental design. (**B**) Weights of BAT and iWAT collected from *Ldhb tm1a* −/− mice or control littermates (WT) that underwent prolonged cold exposure (n = 3 or 12–15 for *Ldhb tm1a* −/− mice or WT, respectively). (**C**,**D**) Western blotting analyses of BAT (**C**) or iWAT (**D**) collected from *Ldhb tm1a* −/− mice or control littermates (WT) that underwent prolonged cold exposure. The analyses were conducted to detect ATGL, LDHB (upper band, indicated with red arrow), CPT2, and UCP1. HSP90 and TUBULIN were used as loading control (n = 4 or 2 for *Ldhb tm1a* −/− mice or WT, respectively). (**E**,**F**) Western blotting analyses (**E**) and quantification (**F**) of BAT or iWAT collected from *Ldhb tm1a* −/− mice or control littermates (WT) that underwent prolonged cold exposure. The analyses were conducted to detect total OXPHOS. HSP90 was used as a loading control (n = 4 or 2 for *Ldhb tm1a* −/− mice or WT, respectively). * *p* < 0.05.

Cold exposure is an effective mechanism to stimulate heat-generating activity in BAT and to boost adaptive metabolism, i.e., to increase energy expenditure, in WAT. Thus, we conducted prolonged cold-exposure experiments (1 w) and collected both BAT and iWAT at the end of treatment (Figure 5A,C,D). As mentioned earlier, while the masses of BAT were similar between the *Ldhb tm1a* −/− mice and the control littermates, *Ldhb tm1a* −/− mice had reduced masses of iWAT (Figure 2E,F). Notably, after one week of cold exposure, the differences observed in iWAT weights diminished (Figure 5B), implying the failure of iWAT utilization in *Ldhb tm1a* −/− mice. In addition, we performed total OXPHOS Western blotting analyses to detect the complex II (CII) subunit SDHB, the CIII-Core protein 2 (UQCRC2), and the CV alpha subunit ATP5A. Compared to the control littermates, the statistical analyses of SDHB and ATP5A revealed significant reductions in iWAT samples collected from *Ldhb tm1a* −/− mice (Figure 5E,F), suggesting a detrimental effect of LDHB loss on OXPHOS.

## 4. Discussion

Glucose is the body’s energy fuel par excellence, producing ATP via anaerobic glycolysis and oxidative phosphorylation. In mammals, lactate is the dominant product of anaerobic metabolism [18,19,20]. Despite the fact that lactate was initially characterized as a waste product, growing evidence has emerged to reveal the essential role of lactate as a major circulating carbohydrate fuel for various organs [23,24]. Recent works using ^13^C-labelled lactate to trace the consumption of lactate have demonstrated that lactate is a major fuel in the TCA cycle in most tissues of the body, except for the brain [23,24]. In normal tissues, lactate is transported into the cell via high-affinity monocarboxylate transporters 1 (MCT1), according to the transmembrane lactate gradient [30]. In cells with high intracellular concentrations of lactate, low-affinity MCT4 is responsible for the transfer and the maintenance of lactate homeostasis [30]. Following absorption, lactate is converted to pyruvate through LDHB to produce ATP [26]. Although a picture is emerging in which lactate serves as a circulating fuel for different cells to uptake and to oxidize, the utilization of lactate by adipocytes, as well as its potential involvement in the regulation of adipocyte functions, remain unappreciated.

Here, we showed that, during in vitro beige adipocyte differentiation, both mRNA and protein levels of *Ldhb*/LDHB were dramatically increased in differentiated SVF preadipocytes isolated from iWAT, compared with undifferentiated control cells. In this setting, LDHB, as well as the ability of beige adipocytes to utilize lactate as a fuel source, might be important for adipogenesis. Consistent with this, *Ldhb tm1a* −/− mice, where LDHB was functionally knocked out in all cells, had smaller masses of iWAT and eWAT when compared to the control littermates. Since the observed difference could have resulted from either an impediment in adipogenesis or an elevated utilization of fatty acids as a fuel source, we evaluated the global energy metabolism of these mice. As the data regarding oxygen consumption (VO_2_), carbon dioxide production (VCO_2_), and RER indicated, while *Ldhb tm1a* −/− mice exhibited a slight preference towards carbohydrates at nighttime, most of the time, they demonstrated no differences in fuel preference, compared to the control littermates. Thus, the more radical possibility is that, at cellular level, the functional loss of LDHB impedes the utilization of lactate as fuel during adipogenic differentiation, and in turn obstructs the accumulation of iWAT and eWAT. Interestingly, the global knockout of *Ldhb* elevated circulating levels of LDH, creatine, and calcium, implying a disrupted global lactate metabolism and potentially disturbed cardiac functions. Besides, as the blood urea nitrogen (BUN) levels of *Ldhb tm1a* −/− mice demonstrated no significant difference when compared with the control littermates, and the urinary protein was not detected in urine samples collected from *Ldhb tm1a* −/− mice (www.cam-su.cn, accessed on 12 May 2021), we speculated that the global knockout of *Ldhb* did not alter kidney function.

Under circumstances of cold exposure, ATs are able to boost adaptive thermogenesis and to maintain body temperature. As recent studies have revealed, in response to cold exposure, lipolysis and fatty acid oxidation in iWAT might be enhanced in order to provide anaplerotic intermediates to the TCA cycle to improve mitochondrial metabolism, thereby producing more energy to adapt to a cold environment [31,32]. Indeed, fatty acids can fuel mitochondrial respiration in adipocytes [10]. The contribution of lactate in this adaptive process has not yet been described. Here we show that, while *Ldhb tm1a* −/− mice had less iWAT mass than their control littermates, the observed difference diminished after 1 week of prolonged cold exposure, suggesting an interruption in the utilization of iWAT in *Ldhb tm1a* −/− mice. In addition, Western blotting analyses, targeting total OXPHOS, revealed reduced levels of SDHB and ATP5A in the iWAT collected from *Ldhb tm1a* −/− mice that underwent cold exposure, implying a disruption of mitochondrial function of beige adipocytes [33]. On the other hand, in terms of BAT collected from *Ldhb tm1a* −/− mice or the control littermates, no differences were observed in weights under normal conditions, weights after prolonged cold exposure, or total OXPHOS levels (ATP5A, UQCRC2, and SDHB). In this regard, the negative effects of *Ldhb* loss were only identified in beige adipocytes, raising the possibility that the fuel sources for thermogenesis are different for beige and brown adipocytes. It is well-known that oxidative phosphorylation of fatty acids is the primary fuel source for BAT thermogenesis [34,35,36,37]. However, beige adipocytes have a higher metabolic plasticity, enabling them to shift between fuel sources, such as glucose [15,38,39]. Recent studies have demonstrated that a highly specialized subtype of beige adipocytes can obtain energy from glycolysis or glucose oxidation, depending on their availability and the internal thermogenic mechanisms [40,41,42]. Here, our findings show that lactate is also important for the thermogenic function of iWAT. Although the present study did not determine neither the detailed mechanisms controlling the observed impediment in iWAT-related responses to cold exposure, the estimated proportion of lactate metabolism in the overall bioenergetic metabolism of AT, nor the contribution of AT to total energy metabolism, our findings highlight the importance of LDHB-enabled lactate conversion in the thermogenesis of beige adipocytes in mice, adding lactate to the known fuel sources. Future studies should focus on using 13-C labeled lactate to elucidate the metabolic pathways and the consequences of lactate in different adipocytes in vitro (white, beige, and brown) and Ats from various physiological conditions in vivo (obese, diabetes, and thermogenesis). Investigations of tissue-specific knockout mouse strains (*Adiponectin-Cre* and *Ucp1-Cre* to drive *Ldhb* knockout in adipocytes and *Ucp1*^+^ adipocytes, respectively) are also required to dissect the adipocyte-type specific function of LDHB-mediated lactate metabolism. In addition, the ways in which lactate treatment would affect the metabolic abilities and pathways of different types of adipocytes should also be addressed by using multiple approaches, including Seahorse analysis, high-throughput sequencing, and a 13-C labeled oxygen consumption rate (OCR) assay.

## 5. Conclusions

We described the upregulation of LDHB during in vitro beige adipocyte differentiation and the reductions in iWAT and eWAT weights in *Ldhb tm1a* −/− mice, implying the involvement of LDHB in adipogenesis. On the other hand, we found that LDHB in iWAT was downregulated upon cold stimulation and recovered during prolonged cold exposure. In addition, the loss of functional LDHB in *Ldhb tm1a* −/− mice impeded the utilization of iWAT during the metabolic adaptation in response to a cold environment. Together, these findings revealed the involvement of LDHB in beige adipocyte differentiation and thermogenesis.

## Figures and Tables

**Table 1:** Primer information.

Primers	Foward	Reverse
*ldhb* (expression)	TGCGTCCGTTG CAGATGAT	TTTCGGAGTCTG GAGGAACAA
*ldhb* (genotyping)	TTCTGTGGGCTCT AAGATGCTCC	WT: ATTCAAATT GCTTGCCAGGTGTT
		KO: CTTCCTCCT ACATAGTTGGCAGT

**Table 2:** Antibody information.

Antibodies	Source	Identifier	Dilution
LDHB	Proteintech	14824-1-AP	1:1000
HSP90	Proteintech	13171-1-AP	1:5000
Total OXPHOS	Abcam	ab110411	1:1000
ATGL	Cell Signaling Technology	2439	1:1000
UCP1	Cell Signaling Technology	72298	1:1000
CPT2	Proteintech	26555-1-AP	1:1000
LDHA	Proteintech	21799-1-AP	1:1000
β-Tubulin	Cell Signaling Technology	2128	1:5000

## Data Availability

Data are contained within the article and Supplementary Materials.

## Supplementary Materials

The following supporting information can be downloaded at: https://www.mdpi.com/article/10.3390/nu15224846/s1, Figure S1: Global knockout of *Ldhb* might disrupt global lactate metabolism and cardiac functions. (A–C) Serum levels LDH (A), calcium (B), and blood urea nitrogen (BUN, C) of *Ldhb tm1a* −/− mice or control littermates (WT) (n = 3 or 12–15 for *Ldhb tm1a* −/− mice or WT respectively). *** *p* < 0.001.
