# Supplementary material for: Lactate Conversion by Lactate Dehydrogenase B Is Involved in Beige Adipocyte Differentiation and Thermogenesis in Mice
Source: Nutrients. 2023 Nov 20;15(22):4846. doi: 10.3390/nu15224846 (PMC10674895; doi:10.3390/nu15224846)
Supplement: Supplementary file 1 [file nutrients-15-04846-s001.zip › nutrients-2679681-supplementary.pdf]

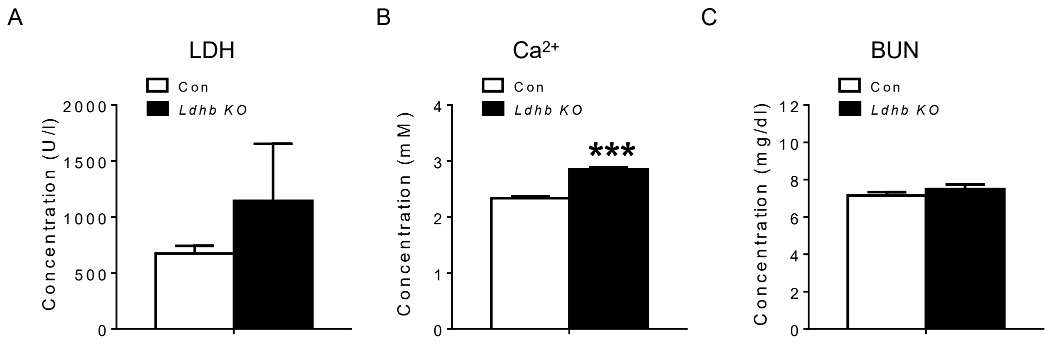

Supplemental Figure S1. Global knockout of *Ldhb* might disrupt global lactate metabolism and cardiac functions. (A–C) Serum levels LDH (A), calcium (B), and blood urea nitrogen (BUN, C) of *Ldhb tm1a -/-* mice or control littermates (WT) (n = 3 or 12–15 for *Ldhb tm1a -/-* mice or WT respectively). *** $p < 0.001$.
